# Ion-mediated interactions between like-charged polyelectrolytes with bending flexibility

**DOI:** 10.1038/s41598-020-78684-6

**Published:** 2020-12-09

**Authors:** Yitong Zheng, Cheng Lin, Jin-Si Zhang, Zhi-Jie Tan

**Affiliations:** 1 Hongyi Honor School, Wuhan University, Wuhan, 430072 China; 2 Department of Physics and Key Laboratory of Artificial Micro and Nano-Structures of Ministry of Education, School of Physics and Technology, Wuhan University, Wuhan, 430072 China; 3 College of Electrical and Photoelectronic Engineering, West Anhui University, Lu’an, 237012 China

**Keywords:** Biological physics, Chemical physics, Statistical physics, thermodynamics and nonlinear dynamics

## Abstract

Ion-mediated interactions between polyelectrolytes (PEs) are crucial to the properties of flexible biopolymers such as nucleic acids and proteins but the effect of PE flexibility on such interactions has not been explicitly addressed until now. In this work, the potentials of mean force (PMFs) between like-charged PEs with different bending flexibility have been investigated by Monte Carlo simulations and a cylindrical confinement around each PE was involved to model two PEs in an array. We found that in the absence of trivalent salt, the PMFs between like-charged PEs in an array are apparently repulsive while the bending flexibility can visibly decrease the repulsive PMFs. With the addition of high trivalent salt, the PMFs become significantly attractive whereas the attractive PMFs can be apparently weakened by the bending flexibility. Our analyses reveal that the effect of bending flexibility is attributed to the increased PE conformational space, which allows the PEs to fluctuate away to decrease the monovalent ion-mediated repulsion or to weaken the trivalent ion-mediated attraction through disrupting trivalent ion-bridging configuration. Additionally, our further calculations show that the effect of bending flexibility on the ion-mediated interactions is less apparent for PEs without cylindrical confinement.

## Introduction

Highly charged polyelectrolytes (PEs) are essential ingredients of many systems such as colloids, polymers and biopolymers^1–9^. Ion-mediated interactions between charged PEs are critical to the structure stability and assembly of colloids^3–7,10–13^, nucleic acids^14–26^ and proteins^27–33^ due to their polyelectrolyte nature. Counterions can electrostatically bind to PEs and can consequently modulate the effective interactions between them. For oppositely charged PEs, ions can screen the electrostatic attractions between the PEs^34,35^, and it was found recently that multivalent salt of high concentration can modulate the intrinsic Coulomb attractions into apparent repulsions^35–37^. For like-charged PEs, the effective interactions have been paid much attention from two decades ago, and such intrinsically repulsive interactions can be weakened by the screening effect of monovalent ions while can be modulated into apparent effective attractions by multivalent ions^38–45^.

Effective interactions between PEs are generally coupled to their binding counterions which is sensitive to system parameters such as the Bjerrum length (or temperature), the radius of PE rods, and the valence and concentration of ions.^45–47^ For example, apparent repulsions between oppositely charged PEs at high symmetrical multivalent salt are attributed to the counterion release due to the approaching of overcharged PEs by multivalent ions^35^, and similar opposite-charge repulsions at high asymmetrical salt can result from the repulsions between undercharged and overcharged PEs by their respective binding counterions^29^. For like-charge attractions mediated by multivalent ions,^38–45^ various mechanisms have been proposed to explain such attraction, including ion bridging effect^38–40^, ion correlations such as Wigner crystal configuration of binding ions^47–54^, and depletion effect^55^.

However, the existing theoretical modeling and computer simulations for understanding ion-mediated interactions were generally focused on rigid linear PEs, either mostly on parallel configurations^17,37,45,46,56,57^ or occasionally on angularly separated configurations of rigid linear PEs^18,46,57–61^. For example, compared with the parallel configuration of two rigid PEs, the angular separation between two rigid PEs at close separations can apparently weaken the like-charge repulsion for very low multivalent salt and can visibly decrease the effective like-charge attraction for high multivalent salt^46,56,57^. However, the overall effective interaction between two rotatable PEs has not been explicitly addressed. Very importantly, PEs such as nucleic acids and proteins are generally flexible linear polymers rather than rigid ones^62–68^. The flexibility of PEs would allow the conformation fluctuation of PE chains^72–76^, and may apparently affect the effective interactions between them. However, the effect of PE flexibility on ion-mediated interactions between like-charged PEs has not been paid attention until now. Given that bending flexibility is important for describing polymer conformations, it is still required to understand the effect of bending flexibility on the effective interactions between like-charged PEs.

In this work, we calculated potentials of mean force (PMFs) between two like-charged PEs with different bending flexibility in trivalent ion solutions and those between two parallel rigid PEs as the reference case, by extensive Monte Carlo simulations. In the limit of least bending flexibility, our calculations reduced to those for two rotatable rigid PEs. Therefore, specifically, we focused on the effects of rotatability and bendability of like-charged PEs on ion-mediated interactions between them, as well as the corresponding microscopic mechanism. In our simulation model, based on the realistic hexagonal configuration of PE arrays and resultant exclusions from adjacent PEs^17,39–41,61,77–81^, a cylindrical confinement with the radius of PE-PE separation around each PE was involved to model two PEs in a PE array. Additionally, our calculations and analyses are also conducted to understand the effective interactions between two free PEs, through removing the cylindrical confinement.

## Model and simulations

### The model system

**Figure 1:**
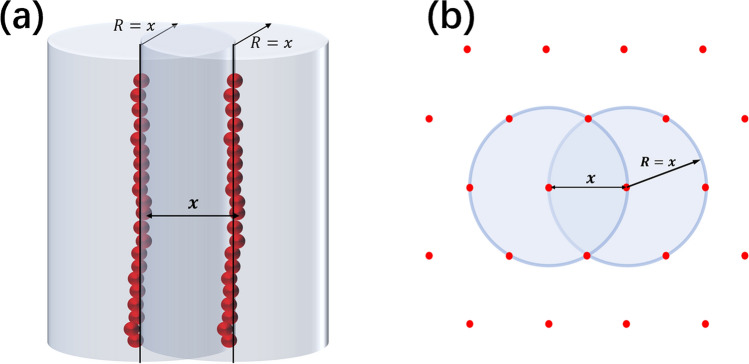
(**a**) Schematic representation of the model system with two like-charged polyelectrolytes (PEs) each of which is confined in a cylindrical boundary with radius *R* = *x*. *x* is the separation between the centers of the central monomers of the two PEs. The PEs are represented by worm-like bead chains with intrinsic bending persistence length *P*. (**b**) Top view of a hexagonal array of PEs. The condensed PEs such as DNAs are generally in a hexagonal array^17,39–41,61,77–81^ and consequently, each PE is spatially restricted (confined) due to the exclusions from its neighboring PEs. To describe such spatial confinement effect, a cylinderical confinement with radius *R* = *x* was involved as shown in the figure.

In this work, we investigated the effective interactions between equally like-charged PEs in salt solutions by MC simulations. In our simulations, two identical negatively charged PEs were immersed in a cubic box, each of which was modeled as a worm-like bead chain with *N* monomers and intrinsic bending persistence length *P*^69–71^. Every monomer bead has a diameter of σ and a negative unit charge of -e (electronic charge). The solvent was modeled as a continuum medium with dielectric constant $\varepsilon_{r}$ = 78, and ions were represented as center-charged spheres with the diameter of σ. In our simulations, the central bead monomers of two PEs were fixed on the x-axis with a separation *x*; see Fig. 1a. Monovalent counterions were added to neutralize the negatively charged PEs and additional 1:1 monovalent salt ions of 20 mM were added as buffer ions^40^. Furthermore, to involve the competition of binding between ions of different valences, three typical concentrations of 3:3 trivalent salt were added in our simulation cells: 0 mM, 0.5 mM, and 5 mM. Trivalent ions were often used as condensing agents for nucleic acids and the used concentrations are typical low, medium, and high concentrations for trivalent ions in nucleic acid condensation^16,39,60,63^. The length of the side of the cubic box was taken as *L* = 62*σ*. The box size is generally kept at least 12 times larger than the Debye–Huckel length in our work to diminish the boundary effect. The periodic boundary condition was applied to the simulation box^35^.

In the model system, there are two types of interactions between all PE monomers and ions: Coulombic interaction and short-ranged excluded-volume repulsion. The Coulombic interaction between two particles of charges *q*_*i*_ and *q*_*j*_ is given by:

1 $$U_{C} = \frac{{q_{i} q_{j} }}{{4\pi \varepsilon \varepsilon_{0} r}} = k_{B} Tl_{B} \frac{{z_{i} z_{j} }}{r},$$

where *T* = 300 K, *l*_*B*_ = 0.714 nm is the Bjerrum length for water at room temperature (300 K), and *r* is the center-to-center distance between the two particles (ions or monomers on PEs). *z*_*i*_ and *z*_*j*_ are the charges of particles *i* and *j* in the unit of e. The short-ranged excluded volume repulsion between particles *i* and *j* is given by a truncated Lenard-Jones potential^80^:

2 $$\begin{array}{*{20}l} {U_{ex} = 4\varepsilon \left( {\frac{{\sigma^{12} }}{{r^{12} }} - \frac{{\sigma^{6} }}{{r^{6} }}} \right) + \varepsilon , } \hfill & {r < 2^{\frac{1}{6}} \sigma ; } \hfill \\ {\quad \,\,\, = 0,} \hfill & {r > 2^{\frac{1}{6}} \sigma . } \hfill \\ \end{array}$$

In our simulations, $\varepsilon$ is set as $\frac{5}{6}$*k*_*B*_*T*^46,56,57^, where *k*_*B*_ is Boltzmann constant and *T* is the absolute temperature in Kelvin.

In our model system, to involve the effect of bending flexibility, each PE is represented by a worm-like bead chain with intrinsic persistence length *P*, and the total bending energy of two PEs is accounted for through persistence length *P*^69,70^:

3 $$U_{b} = k_{B} T\frac{P}{{l_{0} }}\mathop \sum \limits_{i} \left( {1 - \cos \left( {\theta_{i} } \right)} \right),$$

where *l*_0_ = 1.1σ is the original bond length between adjacent monomers. The summation is over all the local bend angles *θ*_*i*_ of the two respective PEs.

Inspired by the realistic aggregate state of the hexagonal assembly of PEs such as DNAs and RNAs^17,39–41,61,77–81^, in our model, each PE was confined in a cylinder with a radius *R* around its initial central axis and such cylindrical confinement is involved to model the exclusion from adjacent PEs in a realistic aggregate state; see Fig. 1a,b. Thus, monomers of each PE are only allowed to move within the cylindrical confinement with radius *R* which was taken as *R* = *x*, a mean value of the PE-PE separation between adjacent PEs in an aggregate (see Fig. 1b). Additionally, we also removed the confinement for typical salt conditions to understand the effective interactions between two free PEs.

### Simulation details

Specifically, in our simulations, the diameter σ of bead monomers and ions was taken as 0.42 nm and the number of monomers in each of PEs was taken as *N* = 21. Moreover, four typical cases for two PEs were used in our simulations: (i) parallel rigid PEs where two PEs were restrained so that the two PEs are kept parallel to the z-axis; (ii) PEs with persistence length *P* = ∞, i.e., two rotatable rigid PEs; (iii) PEs with persistence length *P* = 10 nm, which is comparable to the original length *L* of a PE; and (iv) PEs with persistence length *P* = 2 nm, which is much smaller than *L*.

As described above and shown in Fig. 1, the central monomers were fixed with separation *x*. For each value of *x*, all the monomers except the central monomer of each PEs were allowed to move in the cylinder confinement around the respective axis. In order to improve the sampling efficiency of PEs’ conformations, the pivot rotational move was employed for PEs^69,70^, except for the case of parallel rigid PEs. The Metropolis algorithm was applied to determine the accepting probability of a new configuration of the system^11,69,70^. The process was repeated until equilibrium is reached and until sufficient numbers of conformations in equilibrium were obtained to calculate effective forces and potentials of mean force between the PEs. The system is considered to be in equilibrium when the averaged force acted on one PE is equal to that on the other PE within an error of 0.5%. See Fig. S1 in the Supplementary Material for the curves of the calculated effective forces versus MC steps. In this work, we performed the simulations with our self-developed program instead of the existing well-developed packages. Additionally, our additional simulations show that an apparently larger box size does not have a visible effect on our simulation results; see Fig. S2 in the Supporting Materials.

### Calculating potentials of mean force

Based on the configurations in equilibrium, the force on every monomer of the PEs can be calculated, including the forces induced by Coulombic potential and short-ranged exclusion potential. The force applied on PEs can then be calculated as the summation of forces on their monomers^45,46,56,57^,

4 $$F_{C} = - \mathop \sum \limits_{i} \mathop \sum \limits_{j} k_{B} Tl_{B} \frac{{z_{i} z_{j} }}{{r_{ij}^{2} }};$$

5 $$F_{ex} = - 4\varepsilon \sum\nolimits_{i} {\sum\nolimits_{j} {\left( {12\frac{{\sigma^{11} }}{{r^{12} }} - 6\frac{{\sigma^{5} }}{{r^{6} }}} \right)} ,\quad r < 2^{{\frac{1}{6}\sigma }} } ;$$

6 $$\overline{F} = \frac{1}{2}\left( {F_{C}^{1} + F_{ex}^{1} + F_{C}^{2} + F_{ex}^{2} } \right),$$

where *i* is the index of the monomers on one PE and *j* is the index of other particles, including the monomers on the other PE, counterions, and salt ions. Here, superscript 1 is the force on the first PE and superscript 2 is that on the second PE. The positive direction of the force was defined in such a way that positive force and negative force stands for effective repulsion and attraction respectively, suggesting that the positive direction of the force is opposite for the two PEs. In Eq. (6), the bending energy was not considered since internal forces in one PE do not directly contribute to the PMFs between two PEs. To obtain the PMFs between the PEs, the averaged force on the two PEs was integrated with respect to the center-to-center separation *x* from an outer reference separation *x*_*ref*_,

7 $${\Delta }G = \mathop \smallint \limits_{x}^{{x_{ref} }} \left\langle {\overline{F}} \right\rangle dx,$$

where $\left\langle \cdots \right\rangle$ means the ensemble average over equilibrium configurations. In this work, the outer reference separation was taken as *x*_*ref*_ =8σ.

## Results and discussion

In this section, we showed the calculated PMFs between two like-charged parallel PEs, between two rotatable PEs, and between two bendable PEs in a PE array for different 3:3 trivalent salt concentrations, through involving a cylindrical confinement around each PE to model two PEs in an array. Specifically, we focused on the effects of bendability and rotatability of PEs on the PMFs between two like-charged PEs at different trivalent salt concentrations, and the corresponding microscopic mechanism for two PEs in an array. Finally, we also conducted additional calculations to understand the effective interactions between two free PEs with rotatability and bendability, through removing the cylindrical confinement.

### Potentials of mean force for parallel, rotatable and bendable PEs in arrays

**Figure 2:**
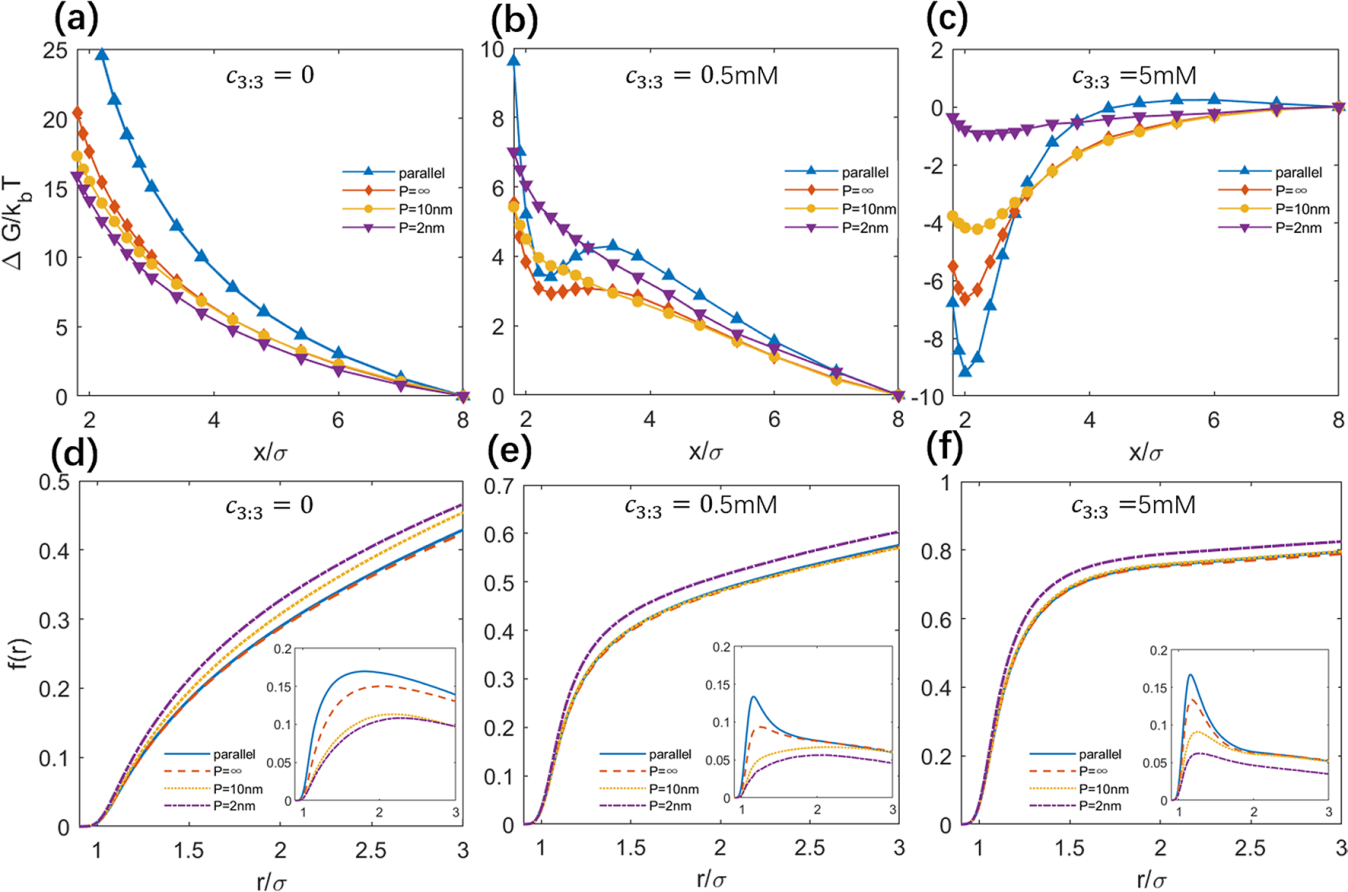
(**a**–**c**) Potentials of mean force between like-charged PEs with different intrinsic persistence lengths *P* for different salt conditions: 3:3 salt concentration *c*_3:3_ = 0 (**a**), 0.5 mM (**b**) and 5 mM (**c**). (**d**–**f**) Charge fraction *f*(*r*) of ions around the PEs at *x* = 8σ for different salts: *c*_3:3_ = 0 (**d**), 0.5 mM (**e**), and 5 mM (**f**). The insets are Δ*f*(*r*) = *f*(*r*)_*x*=2σ_- *f* (*r*)_*x*=8σ_ at the corresponding salt conditions. It is noted that *f*(*r*) and Δ*f*(*r*) include all types of salt ions and counterions; see Eq. (8). Please note that the systems were always in the presence of 20 mM 1:1 salt buffer as described in “Model and simulations” section.

The PMFs for parallel, rotatable and bendable PEs in arrays have been shown in Fig. 2, and the corresponding effective forces have been presented in Fig. S3 in the Supplementary Material.

#### At no trivalent salt

As shown in Fig. 2a, in the absence of trivalent ions, the PMFs between like-charged PEs are dominated by buffer monovalent salt of low concentration and are always repulsive, regardless of the types of PEs. The effective repulsion is the strongest for two parallel like-charged PEs, and when PEs become rotatable, i.e., for the PEs with persistence length *P* = ∞, the effective repulsion between them becomes apparently weakened, which is in accordance with the previous finding that the angular separation decreases effective like-charge repulsion at close separation for low monovalent salt^46,57^. When PEs become bendable, i.e., with the decrease of *P* from ∞ to 2 nm, the repulsive PMFs monotonically decreases. Therefore, the rotatability and bendability both weaken the effective repulsion between like-charged PEs at low buffer monovalent salt.

#### At low trivalent salt

Figure 2b shows the PMF profiles for two parallel, rotatable and bendable PEs at low (~ 0.5 mM) trivalent salt, where monovalent ions of relatively high concentration can play a major role in binding to PEs. With the addition of low trivalent salt, a local minimum appears at close separation in the PMF profile for the parallel PEs while the overall PMF is still positive, suggesting an apparently global effective repulsion with a locally weak attraction for the parallel PEs at low trivalent salt. When two PEs become rotatable, i.e., for the case of *P* = ∞, the local minimum in the PMF profile becomes almost invisible and the global effective repulsion becomes weakened compared with the case of the parallel PEs, suggesting that rotatability of PEs weakens the local attraction at close separation (*x* ~ 2σ) as well as the repulsion in long separation range. When PEs become bendable, i.e., for the PEs with *P* = 10 nm, the PMF between PEs is almost the same as that between PEs with *P* = ∞ in long separation range, while at close separation, the PMF becomes slightly more repulsive. Furthermore, for more bendable PEs, i.e., when *P* is decreased from 10 to 2 nm, the PMF becomes more repulsive than that between PEs with *P* = 10 nm in the whole separation range.

#### At high trivalent salt

At 5 mM trivalent salt concentration, trivalent ions of high concentration can play a dominating role in binding to PEs compared with monovalent salt. As shown in Fig. 2c, with the addition of high trivalent salt, the calculated PMFs are apparently attractive at close separation for two like-charged PEs despite their rotatability and bendability, and the minimums of the PMFs are located at *x* ~ 2σ. For the case of two parallel PEs, the PMF is most negative and its minimum can reach ~ -9*k*_*B*_*T* at *x* ~ 2σ, while the negative PMF increases by ~ 2.5*k*_*B*_*T* at *x* ~ 2σ for the rotatable PEs, i.e., for the PEs with *P* = ∞. When the PEs become bendable, i.e., persistence length *P* decreases from ∞ to 10 nm, the attractive interaction becomes less attractive and the negative PMF increases by ~ 2 *k*_*B*_*T* at *x* ~ 2σ. For the more bendable PEs with *P* = 2 nm, the effective attraction becomes very weak with PMF minimum of ~ − 1 *k*_*B*_*T* at *x* ~ 2σ. The above results indicate that the rotatability and bendability can apparently weaken the effective attraction between like-charged PEs mediated by high trivalent salt.

### Ion distributions and effective separation for parallel, rotatable and bendable PEs in arrays

#### Ion distributions around PEs

Since the PMFs between two PEs are strongly correlated to the ions around the PEs, to understand the effects of PE rotatability and bendability on the PMFs, we calculated the charge fraction distribution *f*(*r*) of ions around PEs as

8 $$f\left( r \right) = - \frac{1}{N}\mathop \smallint \limits_{0}^{r} \mathop \sum \limits_{i} z_{i} c_{i} \left( {\user2{r^{\prime}}} \right)d^{3} \user2{r^{\prime}}.$$

Here, **r'** is the vector of a spatial position from its nearest monomer on the two PEs. *N* is the number of charged monomers (beads) of the two PEs. $z_{i}$ and $c_{i}$ are the ion valence and concentration of ion species *i* at **r'**, respectively.

Figures 2d–f show *f*(*r*)’s at the outer reference separation *x* = 8σ for three salt conditions: no trivalent salt, 0.5 mM trivalent salt, and 5 mM trivalent salt. At no trivalent salt, *f*(*r*)’s of monovalent salt differ slightly for the different types of PEs and follow the order: *f* (*P* = 2 nm) > *f* (*P* = 10 nm) > *f* (*P* = ∞) ~ *f* (parallel), and this is because stronger bending of more bendable PEs would induce stronger electrostatic field nearby and consequently can attract more condensed ions. With the increase of trivalent salt ions which have strong interactions with PEs, *f*(*r*) increases due to the contribution of trivalent ion condensation and lowered ion binding entropy penalty at higher trivalent concentration, and *f* (*r*)’s for different types of PEs follows the order: *f* (*P* = 2 nm) > *f* (*P* = 10 nm) ~ *f* (*P* = ∞) ~ *f* (parallel). Overcharging is not observed at all covered salt conditions shown above possibly because of the relatively low trivalent salt for overcharging and the competition from monovalent salt and counterions, since overcharging generally occurs at very high ion concentration and strong coupling systems^57,76^. Our additional calculations show that the overcharging can be observed if we increase trivalent salt and decrease monovalent ions in our model system (data not shown).

It is more useful to calculate the relative distribution Δ*f*(*r*) between a typical close separation and the outer reference separation by

9 $${\Delta }f\left( r \right) = f_{{x = 2{\upsigma }}} \left( r \right) - f_{{x = 8{\upsigma }}} \left( r \right).$$

Here, *x* = 2σ was used since the PMF minimum is at this separation for high trivalent salt. As shown in Fig. 2, Δ*f*(r) is always positive for three salt conditions, which means that more ions would condense on the two PEs when they approach each other. This is because the PEs with closer separation bring stronger electrostatic field nearby and attract more counterions to condense near PE surfaces. Furthermore, for the four types of PEs, Δ*f*(r) follows the following order: Δ*f* (parallel) > Δ*f* (*P* = ∞) > Δ*f* (*P* = 10 nm) > Δ*f* (*P* = 2 nm). Such order is in accordance with the effective separation between two PEs as shown in the following discussions.

#### Effective separation between PEs

To directly understand the change in PE configurations induced by rotatability and bendability, we introduced an effective separation *D* to characterize the spatial correlation between two PEs, and *D* is given by

10 $$D = \left\langle \frac{1}{N}\mathop \sum \limits_{i = 1}^{i = N} \left| {{\varvec{r}}_{i}^{1} - {\varvec{r}}_{i}^{2} } \right| \right\rangle .$$

where $\left| {{\varvec{r}}_{i}^{1} - {\varvec{r}}_{i}^{2} } \right|$ is the distance between the *i*th monomer on the first PE and the *i*th monomer on the second PE. $\left\langle \cdots \right\rangle$ stands for the ensemble average in equilibrium. As indicated in Eq. (10), *D* actually describes the mean distance between the corresponding monomers belonging to two respective PEs, and *D* = *x* for the simplest case of two parallel PEs and is generally not equal to *x* for rotatable and bendable PEs*.* Here, we introduced *D* because mere distance and angular separations are inadequate for describing the mutual spatial coordinate between two bendable PEs.

**Figure 3:**
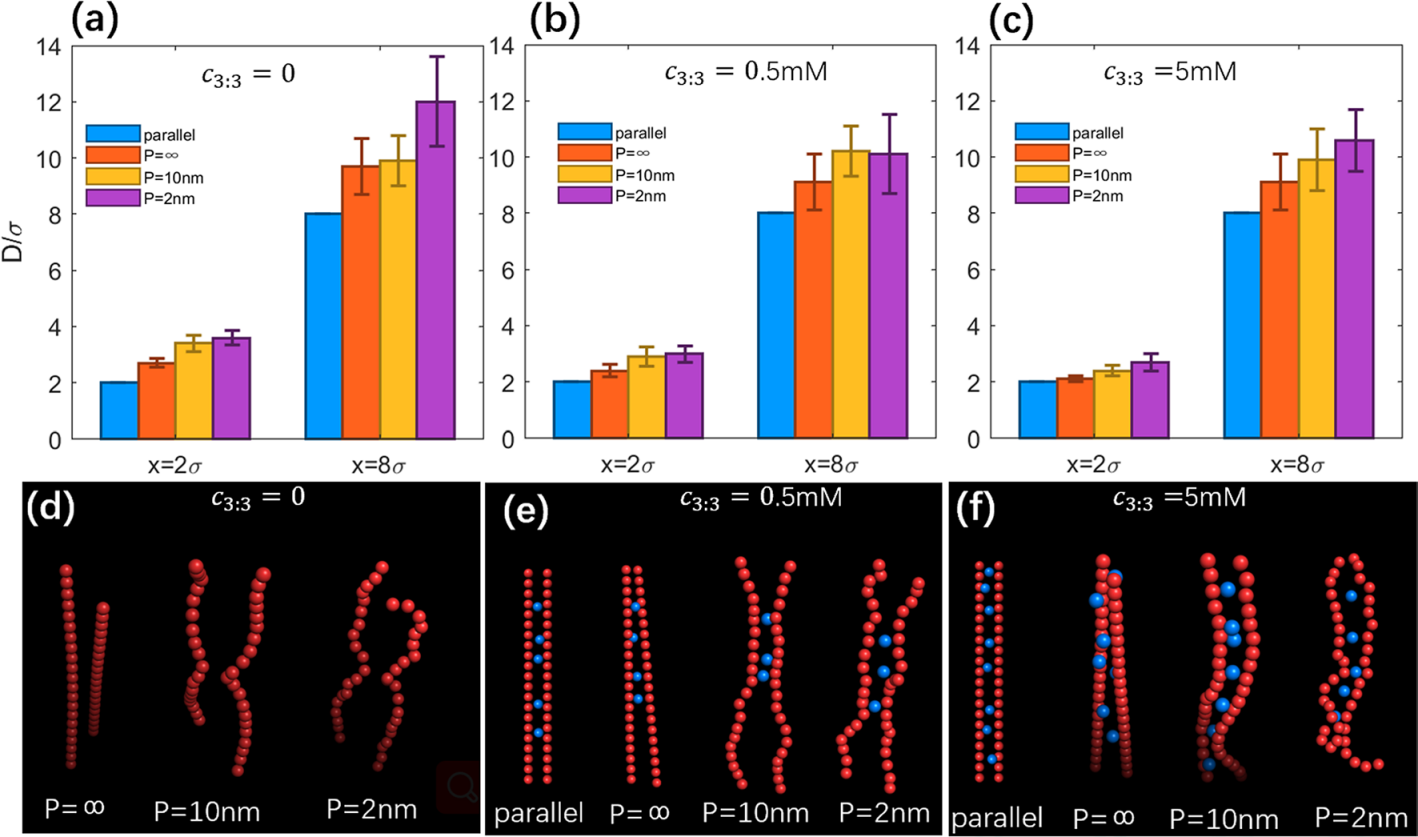
(**a**–**c**) Effective separation *D* between two PEs with different intrinsic persistene lengths *P* at the typical separations (*x* = 2σ and *x*_*ref*_ = 8σ): *c*_3:3_ = 0 (**a**), *c*_3:3_ = 0.5 mM (**b**) and *c*_3:3_ = 5 mM (**c**). Errorbars represent the corresponding standard deviation Δ*D*. (**d**–**f**) Typical conformations of PEs with different persistene lengths *P* for different salt conditions: *c*_3:3_ = 0 (**d**), *c*_3:3_ = 0.5 mM (**e**) and *c*_3:3_ = 5 mM (**f**). Red spheres represent monomers of PEs and blue spheres represent the bridging trivalent ions.

As shown in Fig. 3, at the outer reference separation (*x* = 8σ), *D*’s for different types of PEs are generally larger than *x* and follow the order: *D*(parallel) < *D*(*P* = ∞) < *D*(*P* = 10 nm) < *D*(*P* = 2 nm). When *x* is decreased to a very close separation (*x* = 2σ), *D*’s follow the same order with those at the reference separation while the difference between them is slighter, which may come from the cylindrical confinement at close separation. Such order of the effective separation *D* is fully consistent with the increase of Δ*f*(*r*)’s of condensed ions for the four types of PEs and smaller effective separation *D* between PEs corresponds to larger Δ*f*(*r*) of condensed ions; see Figs. 2 and 3. This is because more rotatable/bendable PEs have stronger conformation fluctuation and tendency to nonparallel/bent configurations and consequently, have larger effective separations *D*. Such conformation fluctuation of two like-charged PEs characterized by effective separation *D*, would definitely affect the effective interactions between them: (i) for low monovalent salt, more rotatable/bendable PEs have larger conformation space to mutually bend/rotate away to avoid strong repulsions between them, and consequently, the bending flexibility can visibly decrease the repulsive PMFs; (ii) with added high trivalent salt, the stronger conformation fluctuation for more rotatable/bendable PEs would cause larger effective separation and consequently weaken the effective trivalent-mediated like-charge attraction, through decreasing PE-PE correlations and the associated binding of trivalent ions, which will be discussed in more details in the following section. Here, it is interesting to show the typical conformations of the different types of PEs for different salt conditions in Figs. 3d–f. At low monovalent salt, the PEs tends to rotate or bend to avoid strong repulsion between them, and when high trivalent salt is added, the PEs prefer to keep close to each other with certain conformation fluctuation for rotatable and bendable PEs.

Therefore, based on the effective separation between PEs, the effects of rotatability and bendability on the PMFs between like-charged PEs in a PE array is attributed to the increased conformation space, and thus the PEs can fluctuate away to decrease the monovalent ion-mediated repulsive PMFs or to weaken the trivalent ion-mediated attractive PMFs through weakening the correlation between PEs and the associated binding of trivalent ions; see also Fig. S4 in the Supplementary Material.

### Bridging ions for parallel, rotatable and bendable PEs in arrays

Following previous studies^38–40^, we calculated the number of “bridging ions” since the bridging ions shared by two like-charged PEs have been previously shown to be responsible for the multivalent ion-mediated attractions between nucleic acid-like PEs^38–41,82–84^. Here, the bridging ions are defined as those counterions whose distances from both of the two PEs are within a cutoff distance *r*_*c*_ and we took *r*_*c*_ = 1.5σ here.

**Figure 4:**
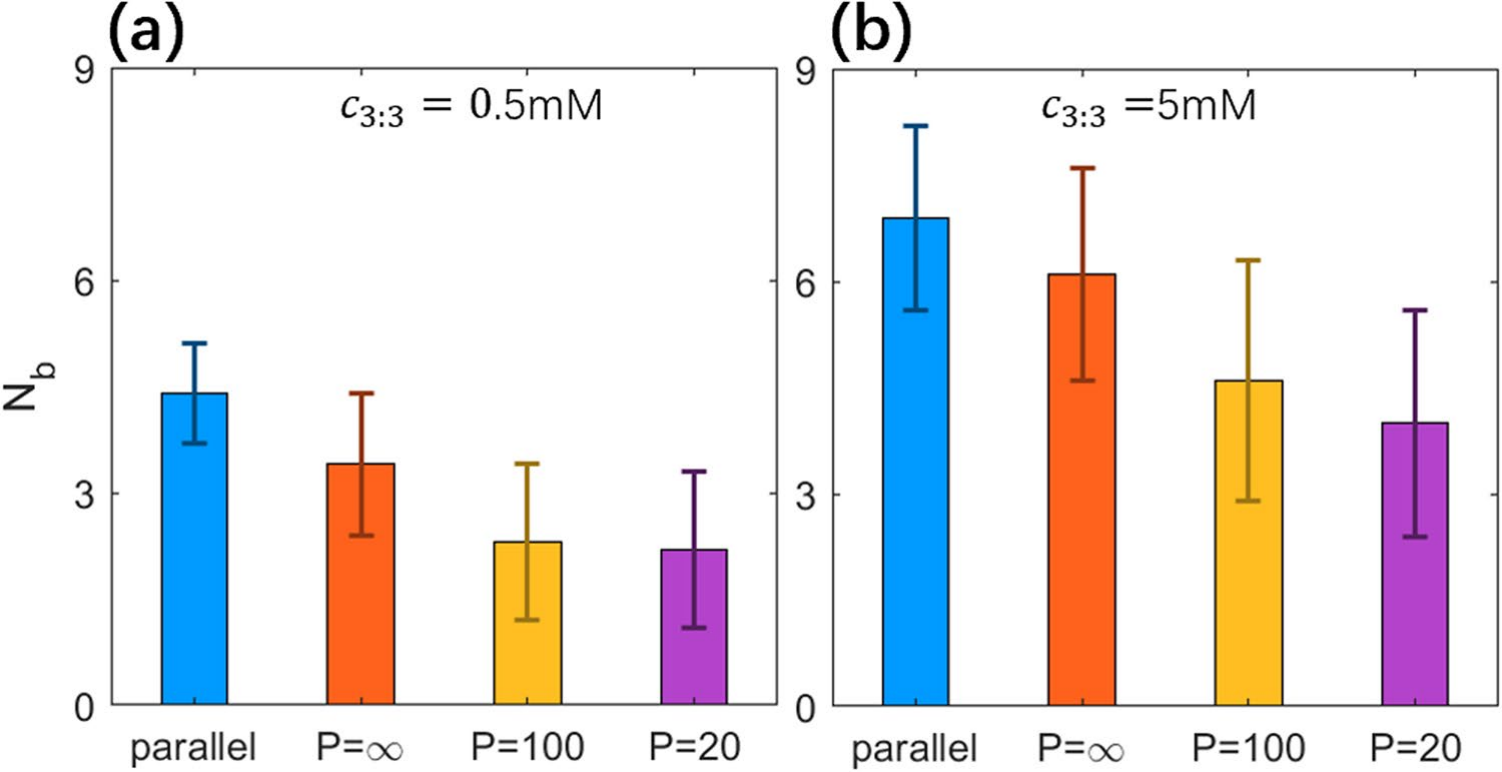
Number *N*_b_ of bridging ions between two PEs for different salt conditions: *c*_3:3_ = 0.5 mM (**a**), *c*_3:3_ = 5 mM (**b**). Errorbars represent the corresponding standard deviation Δ*N*_b_. Please note that the systems were always in the presence of 20 mM 1:1 salt buffer.

As shown in Fig. 3e,f, with the addition of trivalent ions, there are bridging trivalent ions shared by two like-charged PEs. As shown in Fig. 4a, for low trivalent salt (~ 0.5 mM), the number *N*_b_ of bridging ions is small (~ 4.5 in number or ~ 0.3 in charge fraction for the parallel PEs), and *N*_b_ decreases when the PEs become rotatable and bendable, which is corresponding to the weak local attraction at *x* ~ 2σ for rotatable PEs and the disappearance of the weak local attraction for more bendable PEs, as well as the stronger repulsion between PEs with *P* = 2 nm than that between PEs with *P* = 10 nm. Moreover, *N*_b_ and its standard deviation are strongly correlated with the effective separation *D* and its standard deviation, suggesting that the correlation (can be described by effective separation *D*) between two PEs determines the bridging trivalent ions and the corresponding PMFs between them; see also Fig. 2b. As shown in Fig. 4b, for high trivalent salt, compared with low salt, *N*_b_ increases significantly, e.g., *N*_b_ ~ 7 and charge fraction of bridging ions ~ 0.5 for the parallel PEs, resulting in a strong effective attraction between two parallel PEs. When PEs become rotatable and bendable, *N*_b_ decreases due to the mutual conformation fluctuation and the consequent increase of effective separation *D*. For example, *N*_b_ decreases from ~ 6 to ~ 4 when *P* decreases from ∞ to 2 nm. Such apparent decrease in *N*_b_ due to PE bendability would cause the apparent weakening of the effective attraction between like-charged PEs mediated by trivalent ions.

Therefore, for two PEs in an array, the rotatability and bendability of PEs weaken the mutual correlation between two PEs and apparently decrease the number of bridging multivalent ions shared by two like-charged PEs, causing the apparent weakening of effective attraction between like-charged PEs.

### Effect of cylinder confinement: PEs in arrays versus free PEs

**Figure 5:**
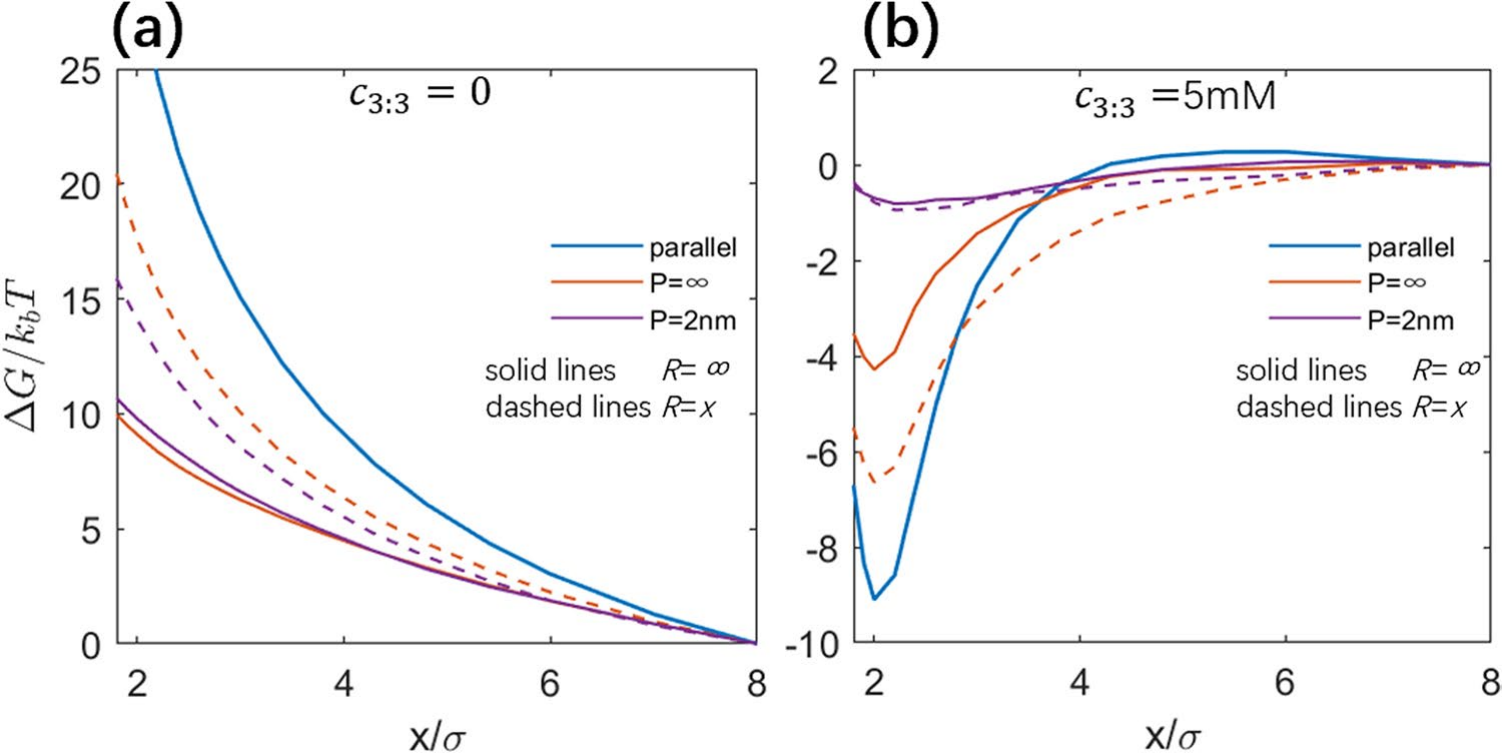
(**a**,**b**) Potentials of mean force between two PEs with different intrinsic persistence lengths *P*: *c*_3:3_ = 0 (**a**), *c*_3:3_ = 5 mM (**b**). Solid lines denote the calculations in the absence of cylindrical confinement and dashed lines denote those for *R* = *x*.

In our model, the cylindrical confinement with radius *R* = *x* was involved to model two PEs in a PE array, which accounts for the exclusions from adjacent PEs in a hexagonal PE array such as DNA and RNA assembly^17,39–41,61,77–81^. It is also interesting to examine the effective interactions between two free PEs through removing the cylindrical confinement, i.e., *R* = *∞*. As shown in Fig. 5a,b, the remove of the cylindrical confinement can have a strong influence on the PMFs between the two like-charged PEs, compared with those of the confinement of *R* = *x*; see Fig. S5 in the Supplementary Material for the corresponding effective forces.

**Figure 6:**
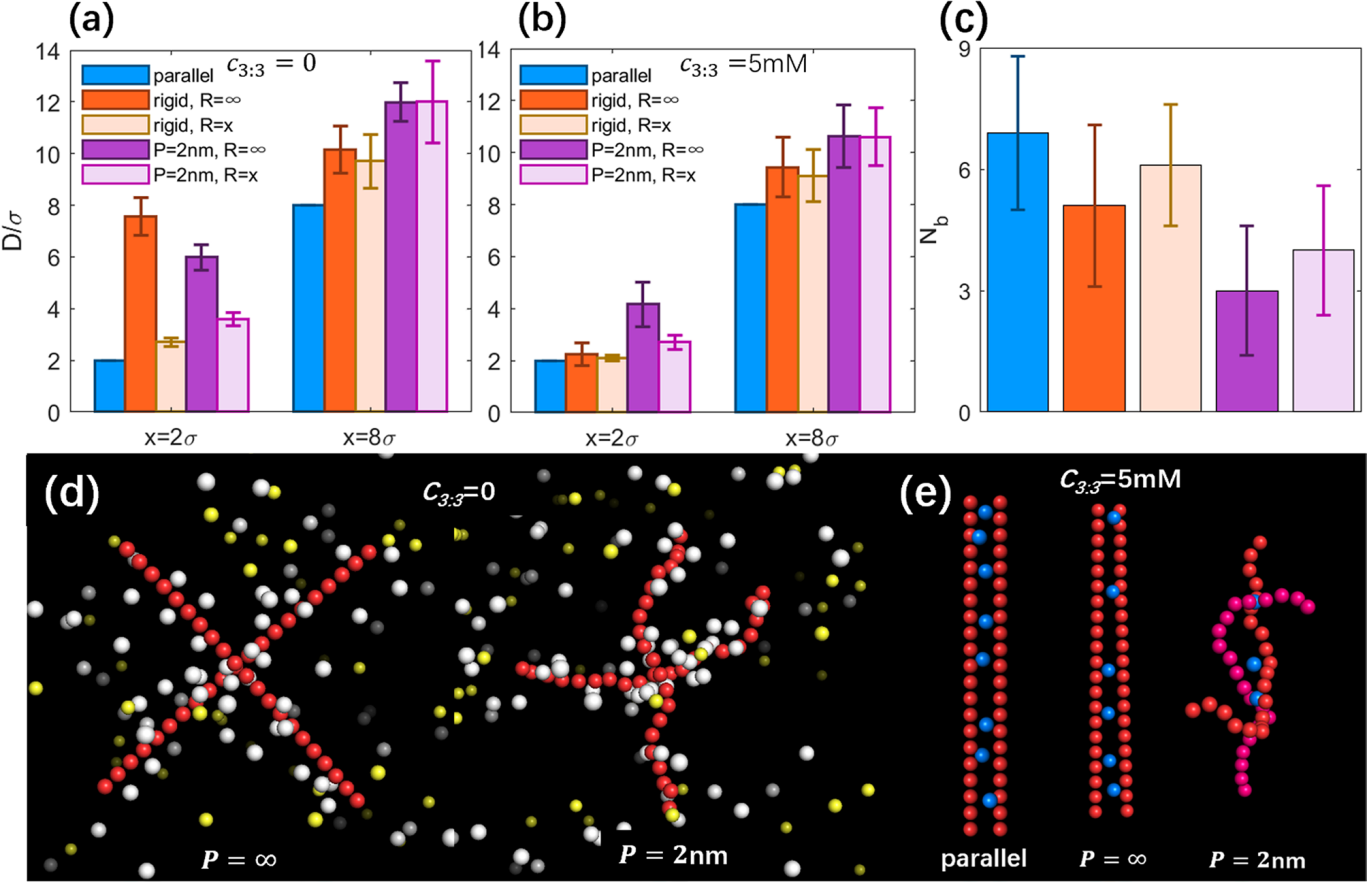
(**a**, **b**) Effective separation *D* between the two PEs for two typical salt conditions: *c*_3:3_ = 0 (**a**) and *c*_3:3_ = 5 mM (**b**), in the absence of the cylinder confinement. Error bars represent the corresponding standard deviation Δ*D*. (**c**) Number *N*_*b*_ of the bridging ions for PEs with different persistence lengths *P* at *c*_3:3_ = 5 mM. Error bars represent corresponding standard deviation Δ*N*_*b*_. Please note that the systems were always in the presence of 20 mM 1:1 salt buffer. (**d**, **e**) Typical conformations of PEs with different persistence lengths *P* in the absence of cylindrical confinement. (**d**) *c*_3:3_ = 0, and white and yellow spheres represent monovalent cations and monovalent anions. (**e**) *c*_3:3_ = 5 mM, and the notation is the same as in Fig. 3. For *P* = 2 nm, the color of one PE is adjusted to pink to make it more distinguishable from the other.

At low monovalent salt (~ 20 mM), the repulsive PMF decreases apparently with the confinement removed. Specifically, the PMF at *x* = 2σ decreases by ~ 8 *k*_*B*_*T* for *P* = ∞ and by ~ 4 *k*_*B*_*T* for *P* = 2 nm. It is also interesting that the repulsive PMF for *P* = ∞ becomes slightly lower than that for *P* = 2 nm in the absence of the confinement. Correspondingly, we examined the effective separation *D* between two rotatable/bendable PEs at typical separation *x* = 2σ. As shown in Fig. 6a, *D* increases apparently, corresponding to the apparent decrease in the PMFs, compared with *R* = *x*; see also Fig. S4 in the Supplementary Material. This is because the remove of the confinement greatly enhances the conformation space for mutual fluctuation of the two rotatable/bendable PEs, and two PEs can become perpendicular to avoid the strong repulsion between them; see Fig. 6d. Compared with *P* = ∞, the PEs with *P* = 2 nm are locally bent and consequently, *D* for *P* = 2 nm is slightly smaller than that for *P* = ∞ and the repulsive PMF is slightly stronger than that for *P* = ∞; see Fig. 6d.

At high trivalent salt (~ 5 mM), when the confinement of *R* = *x* is removed, the PMFs become much less attractive (by ~ 2.5 *k*_*B*_*T* at *x* ~ 2σ) for *P* = ∞ while very slightly less attractive for *P* = 2 nm. Such change in the PMFs is in accordance with the effective separation *D* between the PEs, as shown in Fig. 6b. This is because the PEs can have stronger mutual conformation fluctuation in the absence of the confinement, and the effect of bridging ions can be weakened, especially for less bendable PEs since they are more sensitive to the confinement; see Fig. 6e. For PEs with strong bendability in the presence of the cylindrical confinement, the ion-bridging effect has been strongly disturbed by the conformation fluctuation of PEs due to bending and the effective attraction between them appears weak; see Fig. 6c. Thus, the effect of the furtherly expanded conformation space by the remove of confinement becomes very weak.

From the above, at low monovalent salt, two free PEs with weak bendability repel each other less strongly than those in arrays, and the strong chain bendability can slightly increase the effective repulsion between two free PEs while decrease such repulsion between PEs in arrays. At high trivalent salt, two PEs with weak bendability in arrays can attract each other more strongly than two free ones, while the chain bendability can weaken such effective attraction more strongly for PEs in arrays than for two free ones.

## Conclusion

In summary, in this work, we investigated the ion-mediated interactions between like-charged PEs with different bending flexibility through Monte Carlo simulations. We found that the rotatability and bendability of like-charged PEs have a strong influence on the effective interactions between them and such effect depends apparently on salt conditions. For low monovalent salt without trivalent ions, the effective interactions between like-charged PEs are repulsive due to the screening effect of low-valent ions, and the rotatability and bending flexibility of PEs both weaken such effective like-charge repulsions through increasing the effective separation between PEs due to the increased PE conformational space and the effective mutual repulsion. For high trivalent salt, the like-charged PEs can strongly attract each other through the trivalent ion-bridging effect, while the rotatability and bending flexibility of PEs can significantly reduce such apparent attraction through disrupting trivalent ion-bridging configurations due to the increased PE conformational entropy. Additionally, we found that cylindrical confinement would weaken the overall effects of rotatability and bending flexibility of PEs on the ion-mediated interactions through decreasing conformation space and restricting the conformation fluctuations of PEs. In the present model system, a cylindrical confinement was employed for each PE, which can be a reasonable approximation to model the exclusion to the PE from neighboring PEs in a PE array. If a confinement is employed for the whole system (two PEs), such system can be regarded as the system of two PEs in a crowded environment and is a valuable issue deserved to be investigated separately^19^. Although our simulation model involves some simplifications for realistic PEs such as nucleic acids and proteins, the present work would be very helpful for understanding the ion-mediated interaction between flexible PEs and the structure assembly of flexible PEs.

## Supplementary material

See the Supplementary Material for the following information: (1) The curves of calculated forces versus MC steps to show the simulation equilibriums for typical cases; (2) The calculated mean force as a function of the separation *x* between two PEs for the different types of PEs and different salt conditions in the presence of the cylindrical confinement; (3) The PMFs at typical separation *x* = 2σ versus effective separation *D* between two PEs; (4) The calculated mean force as a function of the separation *x* between two PEs for the different types of PEs and different salt conditions without the cylindrical confinement.

## Supplementary information

Supplementary Information 1.

## Data Availability

The data that support the findings of this study are available from the corresponding author upon reasonable request.
