# Supplementary material for: Ion-mediated interactions between like-charged polyelectrolytes with bending flexibility
Source: Sci Rep. 2020 Dec 9;10:21586. doi: 10.1038/s41598-020-78684-6 (PMC7726156; doi:10.1038/s41598-020-78684-6)
Supplement: Supplementary file 1 — Supplementary Information 1. [file 41598_2020_78684_MOESM1_ESM.pdf]

Supplementary Materials for

Ion-mediated interactions between like-charged polyelectrolytes with bending flexibility

Yitong Zheng[1,2], Cheng Lin[2], Jin-Si Zhang[3], and Zhi-Jie Tan[2*]

[1]*Hongyi Honor School, Wuhan University, Wuhan 430072, China*
[2]*Department of Physics and Key Laboratory of Artificial Micro & Nano-structures of Ministry of Education, School of Physics and Technology, Wuhan University, Wuhan 430072, China*
[3]*College of Electrical and Photoelectronic Engineering, West Anhui University, Lu'an 237012, China*
[*]*zjtan@whu.edu.cn*

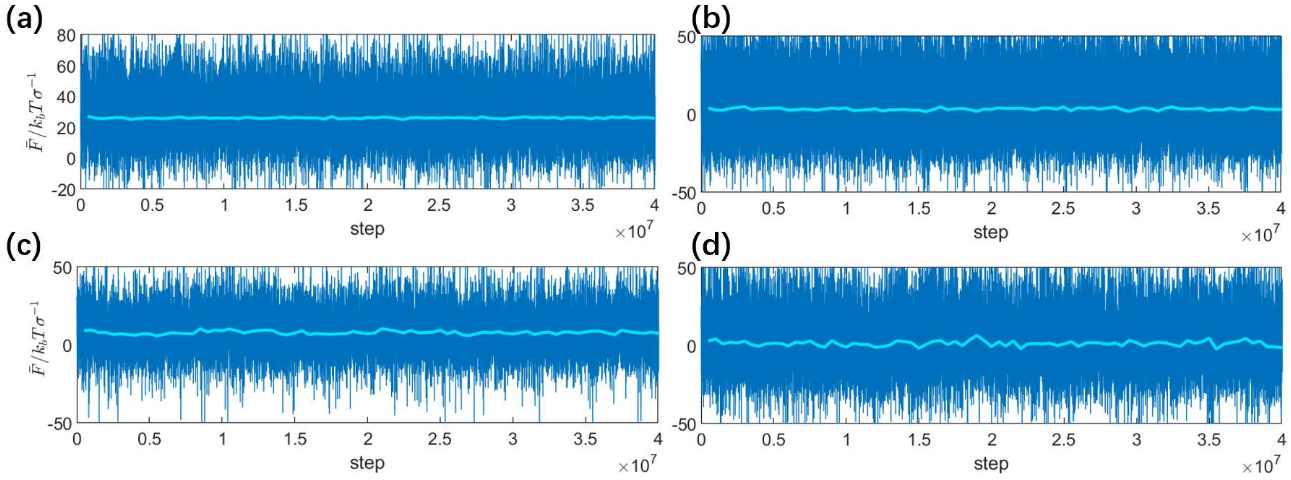

FIG. S1 The calculated forces between two PEs versus MC steps for typical cases at x=2σ: (a) Parallel rigid PEs, $c_{3:3}$=0mM; (b) Parallel rigid PEs, $c_{3:3}$=5mM; (c) P=2nm, $c_{3:3}$=0mM; (d) P=2nm, $c_{3:3}$=5mM. Here, x=2σ is the typical close separation where the PMFs for high trivalent salt have their minimum values. The central light lines represent the average forces over every 5000 steps.

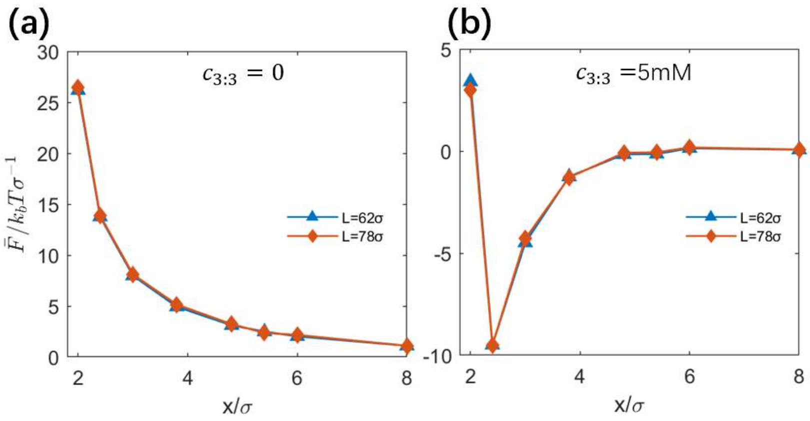

FIG. S2 Averaged forces between two parallel rigid PEs versus separation x at typical trivalent salt concentrations. (a) $c_{3:3}$=0mM; (b) $c_{3:3}$=5mM. Our additional simulations with a larger simulation box (~2 times larger in volume) show the negligible effect of box boundary on the simulation results.

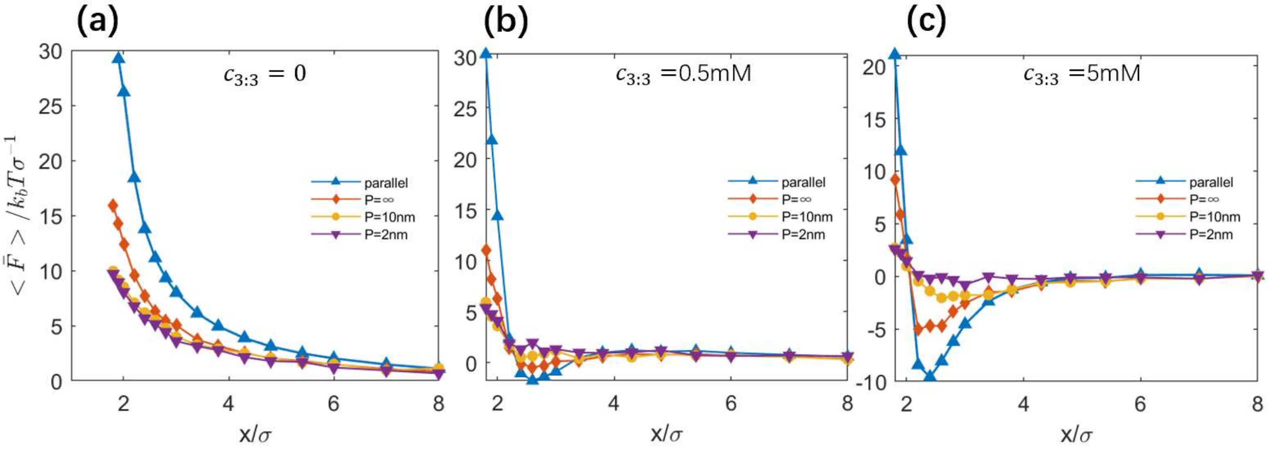

FIG. S3 Averaged forces between the two PEs of four different types versus separation x for three salt conditions: (a) $c_{3:3}$=0mM; (b) $c_{3:3}$=0.5mM; (c) $c_{3:3}$=5mM.

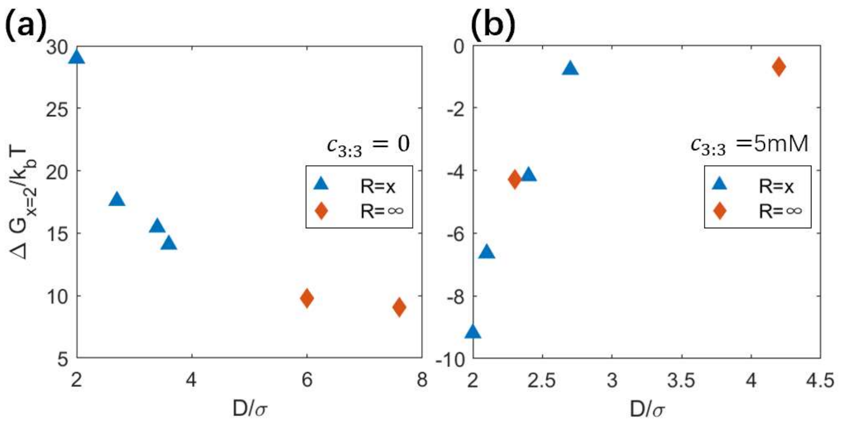

FIG. S4 Potential of mean force at typical separation x=2σ versus effective separation D between two PEs of different types. (a) $c_{3:3}$=0mM; (b) $c_{3:3}$=5mM.

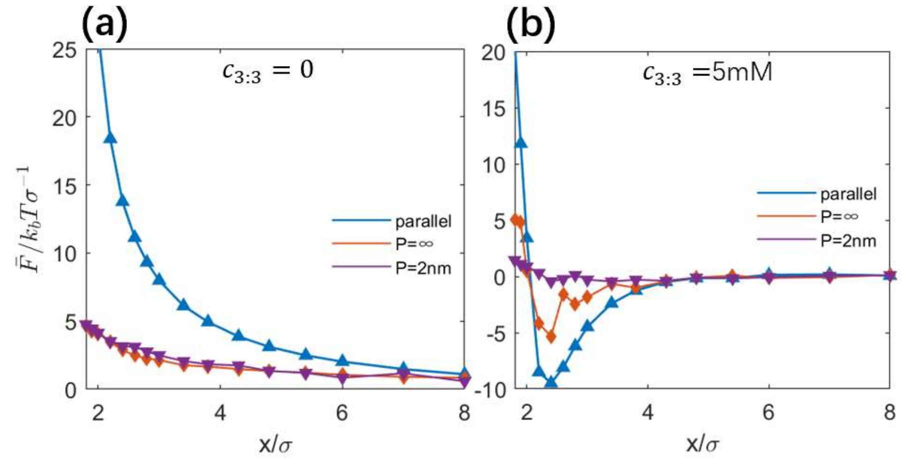

FIG. S5 Averaged forces between the two PEs of four different types versus separation x with no cylindrical confinement. (a) $c_{3:3}$=0mM; (b) $c_{3:3}$=5mM.
